# The therapeutic effects of sodium cromoglycate against influenza A virus H5N1 in mice

**DOI:** 10.1111/irv.12334

**Published:** 2015-12-11

**Authors:** Deping Han, Tangting Wei, Siyi Zhang, Ming Wang, Haiyan Tian, Jinlong Cheng, Jin Xiao, Yanxin Hu, Mingyong Chen

**Affiliations:** ^1^ Key Laboratory of Zoonosis of Ministry of AgricultureCollege of Veterinary MedicineChina Agricultural UniversityBeijingChina; ^2^ Key Laboratory of Veterinary Bioproduction and Chemical Medicine of the Ministry of AgricultureZhongmu Institutes of China Animal Husbandry Industry Co., Ltd.BeijingChina

**Keywords:** influenza A virus, sodium cromoglycate, therapeutic effects

## Abstract

**Objectives:**

To identify the protective role of sodium cromoglycate in mice during influenza virus infection.

**Design:**

H5N1 virus‐infected mice were treated with the mast cell stabilizer sodium cromoglycate (SCG) to investigate its therapeutic effect.

**Sample:**

The nose, trachea and lungs from mice were collected.

**Main outcome measures:**

Virus replication and host responses were determined by plaque assay, quantitative PCR, immunohistochemistry, and histology.

**Results:**

SCG‐treated mice survived better than did PBS‐treated mice after H5N1 virus infection. Mild pathological changes with fewer inflammatory cell infiltration and fewer virus antigens were observed in the nose, trachea, and lungs of SCG‐treated mice on days 3 and 5 post‐infection. However, no significant changes in viral load in the lungs were detected between SCG‐ and PBS‐treated mice. Furthermore, significantly decreased expression of interleukin‐6, tumor necrosis factor‐a, Toll‐like receptor 3, and TIR‐domain‐containing adapter‐inducing interferon‐b was detected in the lungs of SCG‐treated mice, and no higher expression of interferon‐c was detected.

**Conclusion:**

These results suggest that SCG has therapeutic roles in H5N1 virus‐infected mice by alleviating the inflammatory response rather than inhibition of viral replication in the lungs.

## Introduction

Owing to its rapid spread and high pathogenicity, avian influenza virus (AIV) H5N1 has caused severe problems in domestic poultry and humans worldwide. Since the first case of a human infected with highly pathogenic AIV (HPAIV) was reported in Hong Kong in 1997, this has been followed by an outbreak of HPAIV in China in 2004; therefore, AIV has gained significant attention.^1^, ^2^, ^3^ The uncontrolled virus‐induced “cytokine storm” and virus replication in both poultry and humans after HPAIV infection were the main contribution to the acute lung injury and mortality in both humans and animals.^4^, ^5^, ^6^ Studies have shown that the “cytokine storm” produced by the overreacting immune system was responsible for the pathogenesis of H5N1 influenza.^7^ During a cytokine storm in which there is the dysregulation of cytokines, their effects might become excessive and harmful.^8^ The use of anti‐inflammatory doses of corticosteroids to control excessive inflammation might not improve the survival rate of H5N1‐infected mice because of severe side effects.^9^ Furthermore, cytokine and chemokine knockout mice or steroid‐treated wild‐type mice did not have a survival advantage over wild‐type mice following viral challenge.^10^

Mast cells are distributed widely in all tissues throughout the body and are best known for their potent effector functions in allergic diseases.^11^ The involvement of mast cells in tumors and parasitic, bacterial, and viral infections has also attracted widespread attention.^12^, ^13^ Many studies have shown that mast cells play a crucial role in the process of some viral infections, such as dengue virus, human immunodeficiency virus, Newcastle disease virus, and infectious bursal disease virus, by producing many inflammatory molecules including potent proteases, cytokines, chemokines, and arachidonic acid metabolites.^14^, ^15^, ^16^, ^17^, ^18^ Our previous study revealed that mast cells play roles in the pathogenesis of lung injury after H5N1 virus infection by producing proinflammatory mediators including tryptase, histamine, and interferon‐γ (IFN‐γ). In addition, the resulting lung injury was improved by treatment with ketotifen, which inhibits mast cell activation.^19^

Sodium cromoglycate (SCG) can inhibit degranulation and the release of histamine and inflammatory mediators from mast cells. It is used to treat allergic asthma, allergic rhinitis, small intestine ischemia/reperfusion, allergic conjunctivitis, and contact dermatitis;^20^, ^21^, ^22^, ^23^, ^24^ however, its role in the pathogenesis of H5N1 virus infection is unclear. In this study, we investigated whether SCG has protective effects during the initial process of influenza virus infection and the possible mechanism behind any such effects. We show that SCG can improve the mouse survival and respiratory pathological changes. Although viral replication was not inhibited, SCG could regulate the expressions of IL‐6, TNF‐α, TLR3, and TRIF to alleviate the pathological injury to the nose, trachea, and lungs by reducing the inflammatory response.

## Methods

### Animals and cells

Female BALB/c mice aged 6–8 weeks were purchased from Vital River Laboratories (Beijing, China), and the original breeding pairs were from Charles River Laboratories. The mice were housed in independent ventilated cages, and received pathogen‐free food and water. Animal experiments were controlled by the Regulations of Experimental Animals of Beijing Authority and approved by the Animal Ethics Committee of the China Agricultural University.

Madin–Darby canine kidney (MDCK) cell line was provided by the Cell Resource Center of Peking Union Medical College. They were cultured in Dulbecco's minimal essential medium (DMEM; HyClone Laboratories, Logan, UT, USA) containing 10% fetal bovine serum (FBS; HyClone), 100 U/ml penicillin, and 100 μg/ml streptomycin.

### Chemicals

Sodium cromoglycate nasal spray (Vividrin) was purchased from Bausch & Lomb (Germany); 1 ml spray contained 20 mg SCG. Oseltamivir phosphate (Tamiflu; Roche, Basel, Switzerland) was dissolved in 0·85% saline and administered to the mice at a dose of 10 mg/kg body weight, as described previously.^19^

### Virus and challenge

The H5N1 influenza virus (A/chicken/Henan/1/2004, clade 2) used in this study was isolated from infected chicken flocks. This strain has six consecutive basic amino acids at the hemagglutinin (HA) cleavage site, and the receptor binding sites are exactly the same as those in A/Hong Kong/156/97 (H5N1). The virus was isolated from Henan Province, China. The virus was propagated in MDCK cells at 37°C for 48 h, and the viral supernatant was harvested, aliquoted, and stored at −80°C. The 50% lethal dose (LD50) was determined in mice after serial dilution of the stock in phosphate‐buffered saline (PBS). The mice were anesthetized with Zoletil (Virbac, Carros, France) and infected with 3 LD50 in 50 μl intranasally. The body weights of the challenged mice were recorded daily, and the survival rates of the infected and treated mice were calculated on days 3 and 5 post‐infection (pi). Visual grading of clinical changes was performed on days 3 and 5 pi. The standards are as follows: 0, no significant changes; 1–2, inactivity and inappetence; 3–4, inactivity, ruffled hair, inappetence, and emaciation; 5–6; inactivity, ruffled hair, inappetence, emaciation, and labored respiration.

### Treatment trial

The BALB/c mice were selected and divided into the PBS‐, SCG‐, or oseltamivir‐treated groups after infection, eight mice in each group. The survival rate and clinical changes were observed and calculated daily after inoculation. Before the formal test, pre‐tests were performed to verify the optimal dose of SCG that had a therapeutic effect in H5N1 virus‐infected mice. SCG was administered intranasally to anaesthetized mice at a dose of 10, 25, or 50 mg/kg body weight, starting immediately post‐viral challenge, 10 mice in each dose group. The details of the pre‐test and formal test are shown in Table 1.

**Table 1:** Protocols used for pre‐test and formal test

Test	Group	Treatment protocols	
Pre‐test	PBS	PBS: 50 μl intranasally as a control twice daily for 7 days after infection	All mice were anesthetized using Zoletil before intranasal administration
	SCG	Low dose: 10 μl (10 mg/kg) twice daily for 7 days intranasally after infection	
		Moderate dose: 25 μl (25 mg/kg) twice daily for 7 days intranasally after infection	
		High dose: 50 μl (50 mg/kg) twice daily for 7 days intranasally after infection	
Formal test	PBS+virus	PBS: 50 μl as a control twice daily for 7 days intranasally after infection	
	SCG+virus	SCG: 25 μl (25 mg/kg) twice daily for 7 days intranasally after infection	
	Oseltamivir+virus	Oseltamivir: 200 μg in 200 μl (10 mg/kg) twice daily for 7 days intragastrically after infection	

### Plaque assay

Madin–Darby canine kidney cells were cultured in Dulbecco's modified Eagle medium (DMEM) (HyClone Laboratories, UT, USA) containing 10% fetal bovine serum (FBS) (HyClone), 100 U/ml penicillin, and 100 μg/ml streptomycin. Right lung homogenates from individual mice were prepared and diluted 10‐fold in DMEM and added to a monolayer of MDCK cells in semisolid agar containing 0·5‐μg/ml trypsin‐tolylsulfonyl phenylalanyl chloromethyl ketone (TPCK) (Sigma, Beijing, China). Cultures were incubated at 37°C and 5% CO_2_ for 60–72 h, fixed, and stained with 1% crystal violet. The plaque‐forming unit (PFU) was then counted.

### Histology and immunohistochemistry

The right lungs, nose, and trachea were removed from euthanized mice and fixed in 4% neutral formalin at room temperature for 48 h. Serial tissue sections 5 μm thick were obtained after embedding in paraffin. Each slide was stained with hematoxylin and eosin (H&E). Pathological changes were observed and scored under an Olympus microscope (Olympus Optical Co., Ltd.). Criteria for grading changes were previously reported^25^ with some modifications: grade 0 = no obvious pathological changes; grades 1–3 = inflammatory cell infiltration, hemorrhage, cell shedding; grades 4–5 = inflammatory cell infiltration, hemorrhage, vasculitis or bronchiolitis, and cell apoptosis and necrosis; grades 6–10 = severe inflammatory cell infiltration, severe hemorrhage, vasculitis or bronchiolitis, obvious edema, cell apoptosis and necrosis, and microthrombosis.

Tissues sections were stained as described previously^26^ using an anti‐influenza virus nucleoprotein monoclonal antibody (mAb; AA5H; Abcam, Hong Kong, China) at a 1:1000 dilution. Sections were examined by light microscopy (BX41; Olympus, Tokyo, Japan).

### Viral RNA determination by real‐time PCR

Total RNA was prepared from 10 mg tissue from left lungs homogenized in TRIzol reagent (Invitrogen, UT, USA) according to the manufacturer's instructions. DNase I‐treated RNA (0·2 μg) was reverse‐transcribed into cDNA using random or universal primers for influenza A virus (Uni12).^27^ Real‐time PCR was performed to amplify the HA gene of H5N1 influenza virus using the Power SYBR Green PCR Master Mix Kit (ABI, Foster City, CA, USA) and the following primers: forward, 5′‐CGCAGTATTCAGAAGAAGCAAGAC‐3′; and reverse, 5′‐TCCATAAGGATAGACCAGCTACCA‐3′. The reaction was run on an ABI 7500 with the following steps: 95°C for 10 min, and 40 cycles of denaturation at 95°C for 15 s, annealing at 56°C for 30 s, and extension at 72°C for 40 s. Data analyses were performed using the 7500 software (version 2.0; ABI) supplied with the instrument. The copy number of the HA gene was calculated using an HA‐containing plasmid of known concentration as a standard.

### Gene expression

Total RNA was prepared previously, and the RNA was purified using the RNeasy mini kit (Qiagen, USA). The RNA quality and purity were determined by NanoDrop ND‐1000 spectrophotometer at 260/280 nm (NanoDrop Technologies, USA). A total of 0·5 μg of total RNA was transcribed into cDNA with the PrimerScript RT reagent Kit (Takara, Japan) according to the manufacturer's instruction. The expression of tumor necrosis factor‐α (*TNF‐*α), interleukin‐6 (*IL‐6*), *IFN‐*γ, Toll‐like receptor 3 (*TLR3*), TIR‐domain‐containing adapter‐inducing interferon‐β (*TRIF*), and β*‐actin* was detected by real‐time quantitative PCR. The real‐time PCR primers used to amplify *TNF‐*α, *IL‐6*,* IFN‐*γ, *TLR3*,* TRIF*, and β*‐actin* are listed in Table 2. The reactions were carried out with an initial denaturation at 95°C for 10 min followed by 45 cycles of denaturation at 95°C for 15 s, annealing at 50°C for 30 s, and extension at 72°C for 40 s. Gene expression was normalized to that of the control group using the $2^{-\Delta \Delta C_{t}}$ method with β*‐actin* as the internal standard.

**Table 2:** The primers used to detect the gene expressions in the lungs

Cytokines	Primers	
TNF‐α	Forward	5′‐CTGTAGCCCACGTCGTAGC‐3′
	Reverse	5′‐TTGAGATCCATGCCGTTG‐3′
IL‐6	Forward	5′‐AGCCAGAGTCCTTCA‐3′
	Reverse	5′‐TCTTGGTCCTTAGCC‐3′
IFN‐γ	Forward	5′‐ACACTGCATCTTGGCTTTGCAGCT‐3′
	Reverse	5′‐TGAGCTCATTGCATGCTTGGCGCT‐3′
TLR3	Forward	5′‐CCCTTCACCTTTCCG‐3′
	Reverse	5′‐TCATCTAAGCCGTTGG‐3′
TRIF	Forward	5′‐AACCTCCACATCCCCTGTTTT‐3′
	Reverse	5′‐CGGGCACCTGAAATTCCTCA‐3′
β‐Actin	Forward	5′‐GAGACCTTCAACACCCCGC‐3′
	Reverse	5′‐ATGTCACGCACGATTTCCC‐3′

### Statistical analysis

Data were expressed as means ± standard error (SE). The significance of the variability between different groups was determined by two‐way tests of variance using the graphpad prism software (version 5.0). *P* values < 0·05 were considered to be statistically significant.

## Results

### The protective effect of SCG in H5N1‐infected mice

**Figure 1:**
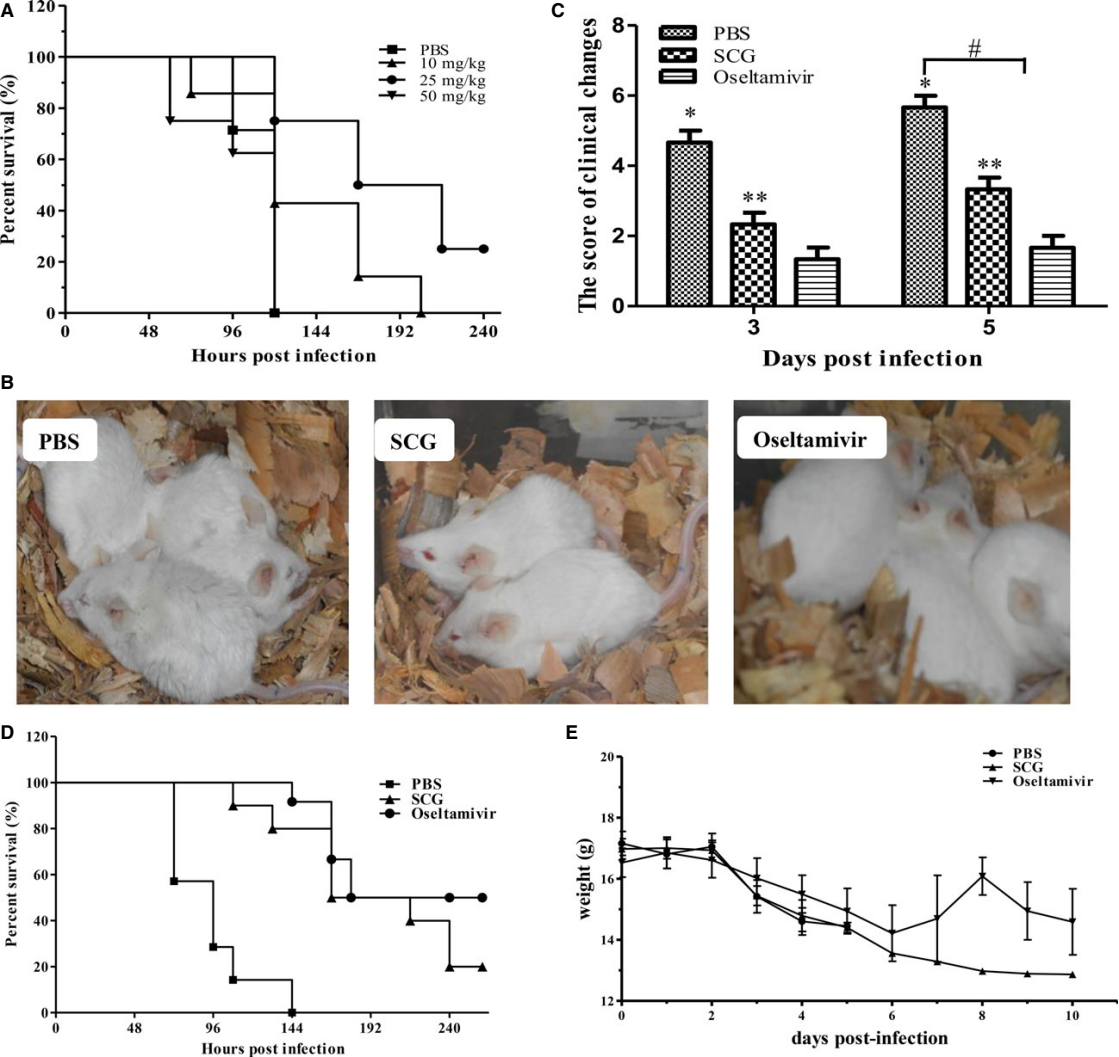
The therapeutic effects of SCG on H5N1 virus infection in mice. (A) The survival rates of H5N1‐infected mice after treatment with different doses of SCG (*n* = 10). (B) The clinical symptoms of H5N1‐infected mice after treatment with oseltamivir or SCG 24 h post‐infection. (C) The scores of clinical changes in H5N1‐infected mice after treatment. (D) The survival rates of H5N1‐infected mice after different treatments (*n* = 10). (E) The changes of body weight in treated mice after H5N1 infection.

To investigate the protective effect of SCG in H5N1‐infected mice, the survival rate, body weight, and clinical signs were recorded. As shown in Figure 1A, 25 mg/kg was the most efficient dose against H5N1 virus (25% survival rate on 240 h pi [hpi]), whereas 10 mg/kg and 50 mg/kg yielded 0·0% survival on 204 and 120 hpi, respectively. Based on the dose–response data described above, we selected the 25 mg/kg dose of SCG for subsequent experiments. PBS‐treated mice presented with clinical signs of inactivity, ruffled hair, inappetence, emaciation, and labored respiration from 24 hpi; however, the mice treated with oseltamivir or SCG showed no obvious clinical signs (Figure 1B). Visual grading of clinical changes was performed (Figure 1C). Compared with the mice in PBS group, mild clinical changes were observed in the mice after SCG and oseltamivir treatment. Unlike the 0·0% survival observed on 144 hpi in PBS‐treated mice, the survival rates of the SCG‐ and oseltamivir‐treated mice were 15% and 22% on 240 hpi (Figure 1D), respectively. No significant differences in body weight were observed between PBS‐, SCG‐, and oseltamivir‐treated mice (Figure 1E). These results suggest that SCG treatment could improve the clinical symptoms and survival of H5N1‐infected mice.

### Virus replication in the lungs

**Figure 2:**
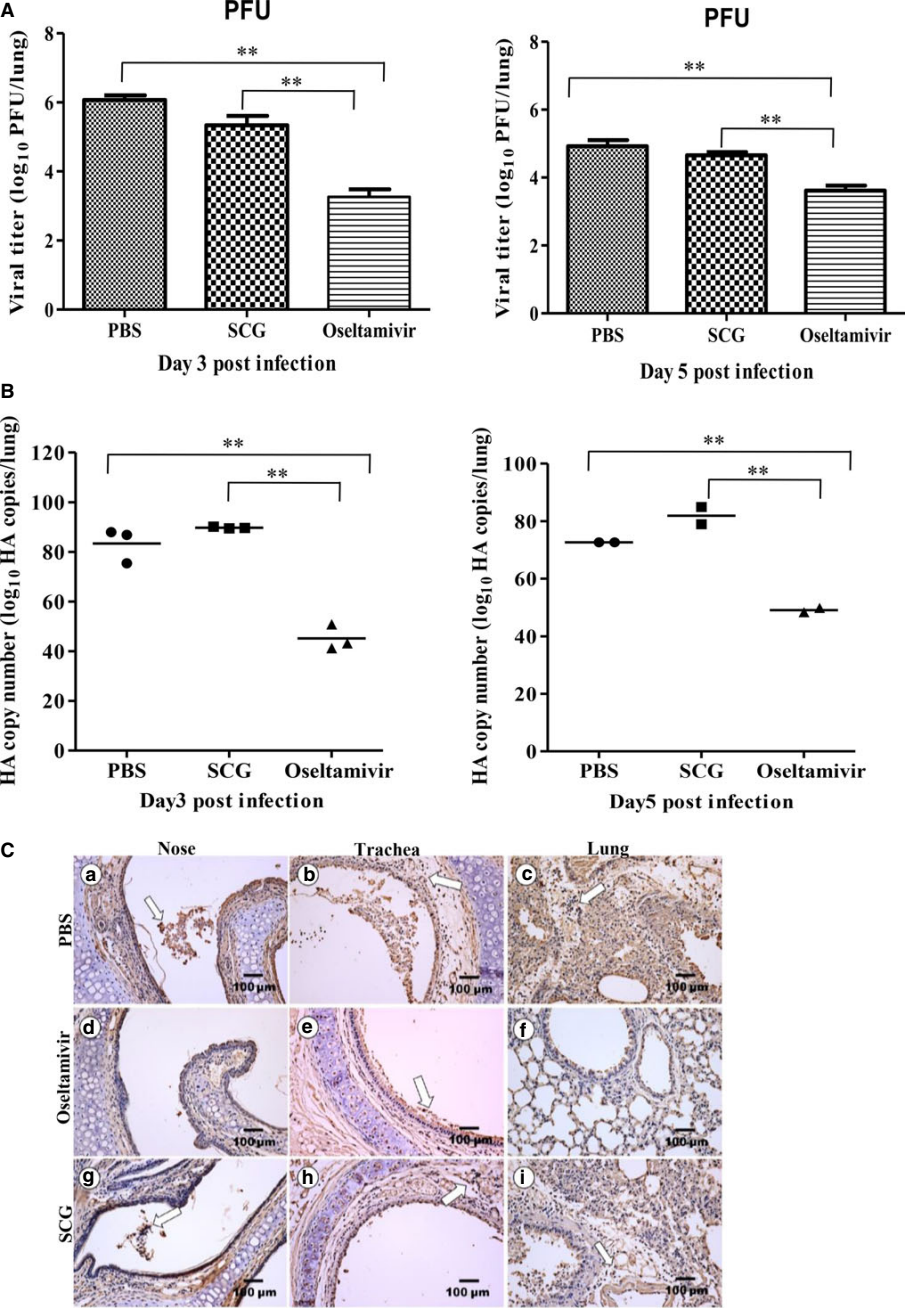
Viral loads in the lungs of H5N1‐infected mice after treatment with oseltamivir or SCG. (A and B) Viral loads in the lungs at the indicated days post‐infection (*n* = 3 per group) were estimated using real‐time PCR (A) or plaque assays (B). The data shown are representative results from three independent experiments. Statistically significant differences between the control and treated groups are indicated by ** (*P *<* *0·01). (C) The distribution of anti‐influenza virus nucleoprotein antibodies in the lungs of H5N1‐infected mice after the indicated treatments on day 5 post‐infection, as determined using immunohistochemistry. Open arrow indicates the positive cells.

To further determine the protective effects of SCG in H5N1‐infected mice, we examined viral replication in the lungs of infected mice. Plaque assays were performed to assess the presence of viral particles in the lung homogenates. On day 3 pi, obvious PFU were present, but there was no difference between homogenates from control mice, infected mice, and SCG‐treated mice. However, significant differences were detected in oseltamivir‐treated mice. Similarly, no significant difference was seen in infected control and SCG‐treated mice on day 5 pi, whereas significantly fewer PFU were present in oseltamivir‐treated mice (*P *<* *0·01) (Figure 2A).

The copy numbers of the hemagglutinin gene were determined using real‐time quantitative PCR to assess viral replication in the lungs. As shown in Figure 2B, no difference was observed between infected control and SCG‐treated mice on days 3 and 5 pi. However, significantly fewer copy numbers were detected in oseltamivir‐treated mice compared with both SCG‐treated and control mice (*P *<* *0·01). After infection, a large number of influenza virus N protein‐positive cells were detected in mucosa epithelial cells, alveolar epithelial cells, lamina propria of the trachea, and inflammatory lymphocytes in H5N1‐infected mice. Although fewer positive cells were observed in the same tissues of SCG‐treated mice, positive cells in the lungs of oseltamivir‐treated mice were only observed in mucosa and alveolar epithelial cells (Figure 2C). These results suggest that SCG treatment did not significantly reduce the viral titer in the lungs of H5N1‐infected mice.

### Pathological changes

**Figure 3:**
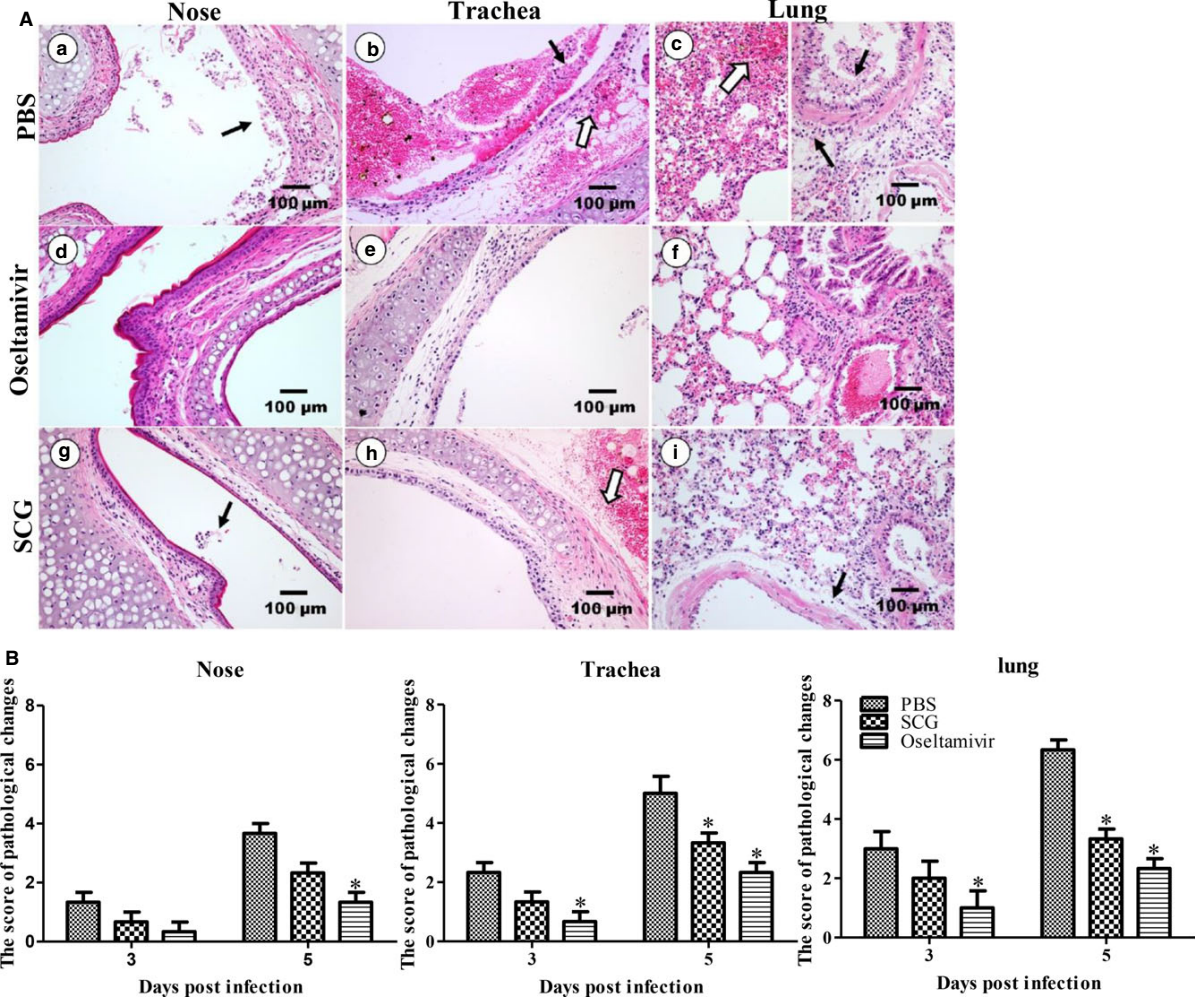
Pathological changes in the respiratory tissues of mice from the different groups. (A) On day 5 post‐infection, severe dropout of epithelial cells, inflammatory cellular infiltration (black arrow), and hemorrhage and edema (open arrow) were observed in the in trachea and lungs of virus‐infected mice (a–c). No obvious injury was observed in oseltamivir‐treated mice post‐infection (d‐f). Mild cell debris, inflammatory cells (black arrow), and hemorrhage (open arrow) were observed in SCG‐treated mice post‐infection (g‐i). (B) The scores of pathological changes in H5N1‐infected mice on days 3 and 5 after treatment.

H5N1‐HPAIV infection can lead to severe lung lesions with the histopathological features of progressive pneumonia. To determine whether the degree of histopathological changes could be improved by SCG treatment, the lungs of mice in each group were examined on days 3 and 5 pi. On day 3 pi, hyperemia and hemorrhage in the trachea and lungs, a small amount of inflammatory cell infiltration into the nose and trachea, peribronchiolitis, and desquamation of the bronchia epithelial cells were observed in virus‐infected mice. In SCG‐treated mice, no obvious lesions were visible in the nose; there were mild hemorrhage in the submucosa of the trachea, and hyperemia and hemorrhage in the lungs with a small amount of inflammatory cell infiltration. No significant pathological changes were observed in oseltamivir‐treated mice, with only mild hyperemia and hemorrhage in the lungs. On day 5 pi, severe pathological changes were observed in virus‐infected mice, which presented as a large number of inflammatory cells and epithelial exfoliation in the nose, hemorrhage, edema, and inflammatory cell infiltration in the lamina propria of the trachea, massive hemorrhage, several erythrocytes, and inflammatory cells in alveolar cavity, and necrosis and falloff of the bronchia epithelial cells with significant inflammatory cell infiltration (Figure 3A). In contrast, lesser lesions were observed in SCG‐treated mice, which exhibited a small number of inflammatory cells in the nose, hyperemia and hemorrhage in the outer membrane of the trachea and lungs, and a widened gap around the bronchia that was infiltrated with edema fluid and inflammatory cells (Figure 3A). In oseltamivir‐treated mice, there were no obvious lesions in the nose, a small number of necrotic epithelial cells, edema, a small number of inflammatory cells in the lamina propria of the trachea, mild hemorrhage, and necrotic epithelial cells in the bronchia in the lungs (Figure 3A). No obvious pathological changes were observed in only PBS‐ or SCG‐treated mice without H5N1 infection. After visual grading of pathological changes, the SCG could alleviate the pathological score compared with PBS‐treated mice after H5N1 infection (Figure 3B). These results suggest that SCG treatment could significantly reduce the inflammatory response and lesions in the nose, trachea, and lungs of H5N1‐infected mice. Therefore, it has the potential to improve the clinical outcome of H5N1 infection.

### Expression of IL‐6, TNF‐α, IFN‐γ, TLR3, and TRIF in the lungs

**Figure 4:**
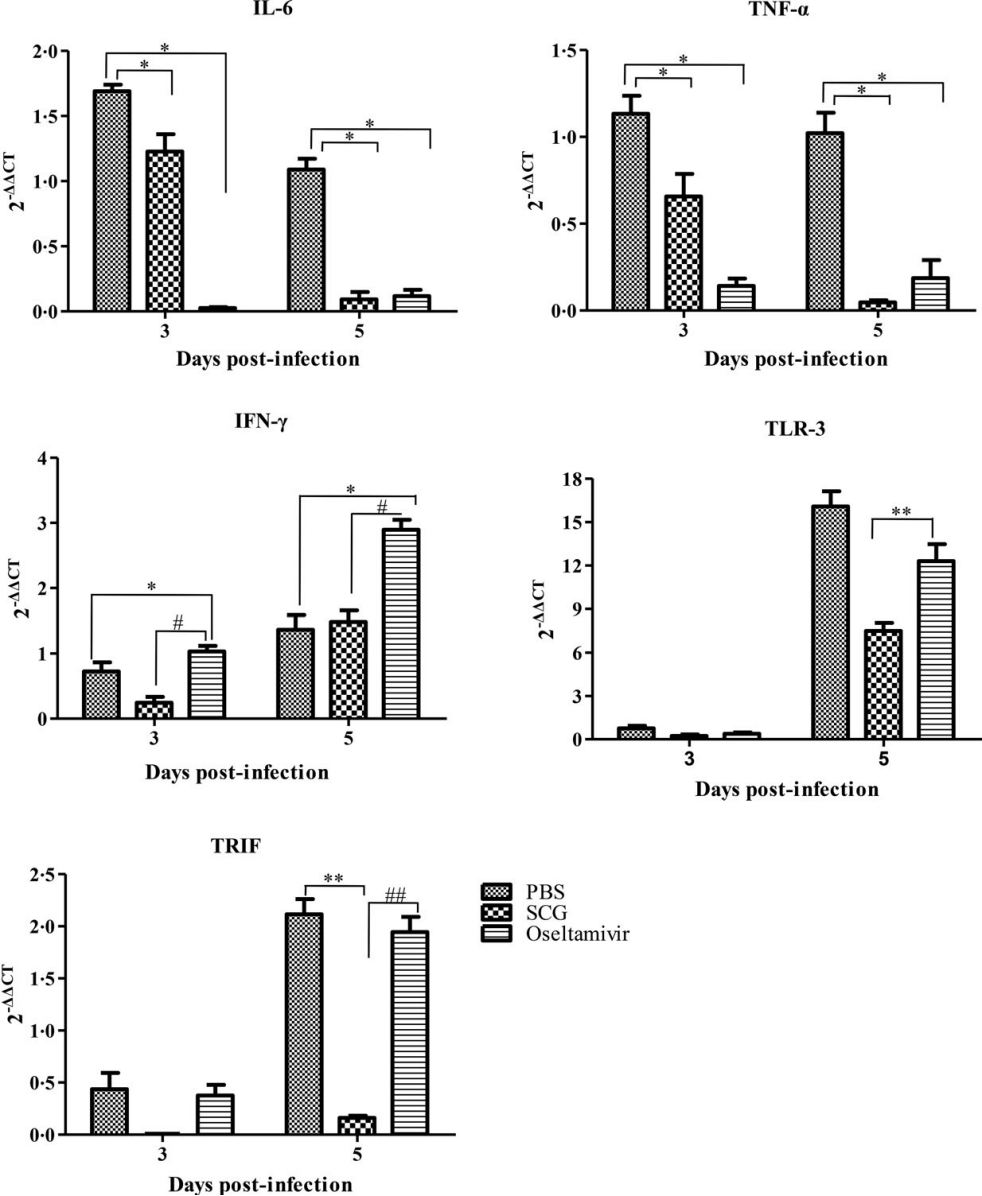
The expression of *IL‐6*,*TNF‐*α, *IFN‐*γ, *TLR3*, and *TRIF* in the lungs of mice in the different treatment groups on days 3 and 5 post‐infection was determined using real‐time PCR. The results are presented as means ± SDs of triplicate samples from three independent experiments. * *P *<* *0·05 and ** *P *<* *0·01 compared with PBS group after infection; # *P *<* *0·05 and # *P *<* *0·01 compared with SCG group after infection.

To further investigate whether SCG treatment could reduce the inflammatory response and possible mechanisms in H5N1‐infected mice, the expression of the genes *IL‐6*,* TNF‐*α, *IFN‐*γ*, TLR3*, and *TRIF* in the lungs was examined using real‐time quantitative PCR. As shown in Figure 4, SCG treatment reduced the expression of *IL‐6* and *TNF‐*α from day 3 to day 5 pi, and the difference was significant between days 3 and 5 pi (*P *<* *0·05). Continuously lower expression of *IL‐6* and *TNF‐*α was detected in the lungs of oseltamivir‐treated mice, and the difference was significant on days 3 and 5 pi compared with virus‐infected control mice (*P *<* *0·05). There were no significant differences in *IFN‐*γ expression in SCG‐ and PBS‐treated mice, but significant differences were detected in oseltamivir‐treated mice (*P* < 0·05). Different from these in PBS‐treated mice, the gene expression of *TLR3* and *TRIF* in SCG‐treated mice was not significantly increased (*P *<* *0·01). These observations were consistent with the histopathological changes described above, which suggested that SCG treatment could significantly reduce the inflammatory response in the lungs of H5N1‐infected mice by downregulation of TLR3 and TRIF expression. Therefore, SCG has the potential to improve survival during H5N1 infection.

## Discussion

Highly pathogenic influenza A virus infection could induce acute lung injury with an accompanying severe inflammatory response, which is considered to be the main cause of death.^28^, ^29^, ^30^ Currently, oseltamivir is the first choice treatment, which has a significant effect on influenza A virus replication and inflammatory cytokine production, and can obviously improve the survival rate. However, an increasing number of drug‐resistant viruses have been discovered recently owing to the enormous genetic variation of influenza virus.^31^, ^32^, ^33^, ^34^, ^35^, ^36^, ^37^ Because of the amplified inflammatory response after H5N1 infection, anti‐inflammatory drugs have been considered to treat humans exposed to the influenza A virus. A previous study found that orthotopic autologous liver transplantation could induce acute lung injury and that the administration of cromolyn sodium or ketotifen significantly inhibited the activation of mast cells and downregulated proinflammatory cytokine levels.^38^ Our previous studies demonstrated that mast cells are involved in lung injury in H5N1‐infected mice and that ketotifen treatment could significantly attenuate the pathological changes in the lungs.^19^ However, the present study is the first to demonstrate therapeutic effects of SCG against influenza A virus H5N1 in mice. Specifically, SCG treatment resulted in improved survival rates and clinical symptoms, alleviated lung lesions, and reduced levels of proinflammatory cytokines.

In this study, no significant difference was found in the viral titers in the lungs of SCG‐ and PBS‐treated mice on days 3 and 5 pi, which suggests that SCG did not inhibit H5N1 virus replication. In contrast, significant differences in the viral titers were detected between the oseltamivir group and the other two groups. Therefore, these results suggest that the SCG and oseltamivir act via different protective mechanisms because oseltamivir could inhibit viral replication whereas SCG only inhibited mast cell activation. Although virus replication was uninhibited in the lungs after H5N1 infection, but the clinical symptoms and the survival rates were improved after SCG treatment. The results indicate that SCG could protect mice from H5N1 virus infection by regulating the response of the mast cells. Moreover, compared with oseltamivir, the therapeutic effect of SCG was not as effective against highly pathogenic AIV H5N1 infection. This suggests that combination drug therapy might be the choice to delay the emergence of drug‐resistant strains and improve the therapeutic effect.

Analysis of the respiratory pathological changes after H5N1 infection revealed that there was significant inflammatory cell infiltration in H5N1 virus‐infected mice, whereas fewer pathological changes were observed in SCG‐treated mice. This suggests that SCG could stabilize mast cells to reduce the release of cytokines and chemokines and alleviate inflammatory cell infiltration in the lungs. These results further suggest that mast cells are involved in the pathogenesis of H5N1 virus infection and that preventing aberrant inflammation could protect the mice from mortality. Previous studies reported that cytokine dysregulation contributed to the severity of influenza H5N1 virus infection.^39^, ^40^ Consistent with those, the current results in our report further support that an atypical immune response, rather than uncontrollable virus replication, resulted in acute mortality after highly pathogenic H5N1 virus infection.

Cytokines play important roles in innate and adaptive immune responses during virus infection, particularly for eliminating the virus and protecting the host during the early infection period. However, the overexpression of these cytokines causes a variety of complications and pathological injuries. Previous studies reported that TNF‐α and IL‐6 could aggravate the clinical symptoms and histopathological changes of influenza virus‐infected mice.^41^, ^42^ We hypothesize that SCG could stabilize the membrane of mast cells, which inhibits the release of inflammatory mediators and alleviates the inflammatory response induced by H5N1 infection. In the current study, increased expression of TNF‐α and IL‐6 in PBS‐treated mice was detected on day 3 pi, and further increased expression was found on day 5 pi, which involved in the inflammatory lung injury. In contrast, the expression of TNF‐α and IL‐6 was decreased significantly on days 3 and 5 pi in SCG‐treated mice, which suggests that SCG could improve the mortality of virus‐infected mice by preventing the overproduction of proinflammatory cytokines. Many studies have found that mast cells could be involved in virus infection using TLR3, RIG‐I, and MDA5 to sense viral RNA.^43^, ^44^, ^45^ In our study, the expression of TLR3 and TRIF in the lungs of SCG‐treated mice decreased, which suggested that SCG might have some roles in the TLR3 pathway in mast cells during H5N1 infection. Similar to PBS‐treated mice, no increase in IFN‐γ in SCG‐treated mice was observed, which suggests that SCG could not inhibit virus replication, and it is inconsistent with the results of plaque assay. However, different from SCG, oseltamivir could inhibit the virus replication and increase the antiviral cytokine IFN‐γ expression, further indicating that there are different mechanisms of anti‐H5N1 virus infection between these two drugs. The reduced expression of TNF‐α and IL‐6 in SCG‐treated mice resulted in fewer respiratory pathological changes and milder clinical symptoms that were significantly different from those in H5N1 virus‐infected mice. Therefore, we concluded that SCG protected H5N1 virus‐infected mice by reducing proinflammatory cytokine production rather than inhibiting virus replication.

## Conclusion

In summary, SCG protected mice effectively from death after H5N1 infection by alleviating inflammatory injury via its function as an inhibitor of mast cell degranulation. These data provide a novel approach for combating highly pathogenic influenza virus infection.

## References

[1] Claas EC , Osterhaus AD , van Beek R *et al* Human influenza A H5N1 virus related to a highly pathogenic avian influenza virus. Lancet 1998; 351:472–477. 9482438 10.1016/S0140-6736(97)11212-0

[2] Yuen KY , Chan PK , Peiris M *et al* Clinical features and rapid viral diagnosis of human disease associated with avian influenza A H5N1 virus. Lancet 1998; 351:467–471. 9482437 10.1016/s0140-6736(98)01182-9

[3] To KF , Chan PK , Chan KF *et al* Pathology of fatal human infection associated with avian influenza A H5N1 virus. J Med Virol 2001; 63:242–246. 11170064 10.1002/1096-9071(200103)63:3<242::aid-jmv1007>3.0.co;2-n

[4] Us D . Cytokine storm in avian influenza. Mikrobiyol Bul 2008; 42:365–380. 18697437

[5] Lipatov AS , Andreansky S , Webby RJ *et al* Pathogenesis of Hong Kong H5N1 influenza virus NS gene reassortants in mice: the role of cytokines and B‐ and T‐cell responses. J Gen Virol 2005; 86:1121–1130. 15784906 10.1099/vir.0.80663-0

[6] Burggraaf S , Karpala AJ , Bingham J *et al* H5N1 infection causes rapid mortality and high cytokine levels in chickens compared to ducks. Virus Res 2014; 185:23–31. 24657784 10.1016/j.virusres.2014.03.012 PMC7127704

[7] Carter MJ . A rationale for using steroids in the treatment of severe cases of H5N1 avian influenza. J Med Microbiol 2007; 56:875–883. 17577050 10.1099/jmm.0.47124-0

[8] Lam WY , Yeung AC , Chu IM , Chan PK . Profiles of cytokine and chemokine gene expression in human pulmonary epithelial cells induced by human and avian influenza viruses. Virol J 2010; 7:344. 21108843 10.1186/1743-422X-7-344 PMC3002310

[9] Chen H , Smith GJ , Li KS *et al* Establishment of multiple sublineages of H5N1 influenza virus in Asia: implications for pandemic control. Proc Natl Acad Sci U S A 2006; 103:2845–2850. 16473931 10.1073/pnas.0511120103 PMC1413830

[10] Chan MC , Cheung CY , Chui WH *et al* Proinflammatory cytokine responses induced by influenza A (H5N1) viruses in primary human alveolar and bronchial epithelial cells. Respir Res 2005; 6:135. 16283933 10.1186/1465-9921-6-135 PMC1318487

[11] Bradding P , Walls AF , Holgate ST . The role of the mast cell in the pathophysiology of asthma. J Allergy Clin Immunol 2006; 117:1277–1284. 16750987 10.1016/j.jaci.2006.02.039

[12] Sundstrom JB , Little DM , Villinger F , Ellis JE , Ansari AA . Signaling through Toll‐like receptors triggers HIV‐1 replication in latently infected mast cells. J Immunol 2004; 172:4391–4401. 15034054 10.4049/jimmunol.172.7.4391

[13] Gaboury JP , Johnston B , Niu XF , Kubes P . Mechanisms underlying acute mast cell‐induced leukocyte rolling and adhesion *in vivo* . J Immunol 1995; 154:804–813. 7814884

[14] Metcalfe DD , Baram D , Mekori YA . Mast cells. Physiol Rev 1997; 77:1033–1079. 9354811 10.1152/physrev.1997.77.4.1033

[15] Wang D , Xiong J , She R *et al* Mast cell mediated inflammatory response in chickens after infection with very virulent infectious bursal disease virus. Vet Immunol Immunopathol 2008; 124:19–28. 18342956 10.1016/j.vetimm.2008.01.005

[16] King CA , Marshall JS , Alshurafa H , Anderson R . Release of vasoactive cytokines by antibody‐enhanced dengue virus infection of a human mast cell/basophil line. J Virol 2002; 74:7146–7150. 10888655 10.1128/jvi.74.15.7146-7150.2000 PMC112233

[17] Meylan PR , Tam EK , Kornbluth RS , Richman DD . HIV infectivity is not augmented by treatment with trypsin, Factor Xa or human mast‐cell tryptase. AIDS 1992; 6:128–130. 1543555 10.1097/00002030-199201000-00019

[18] Sun Q , Wang D , She R *et al* Increased mast cell density during the infection with velogenic Newcastle disease virus in chickens. Avian Pathol 2008; 37:579–585. 19023756 10.1080/03079450802499092

[19] Hu Y , Jin Y , Han D *et al* Mast cell‐induced lung injury in mice infected with H5N1 influenza virus. J Virol 2012; 86:3347–3356. 22238293 10.1128/JVI.06053-11 PMC3302317

[20] Lin YY , Chou YL , Chu YH , Wu CC , Wang JY , Wang HW . Effects of cromolyn sodium on isolated rat's trachea. Allergy Rhinol (Providence) 2011; 2:e46–e50. 22852116 10.2500/ar.2011.2.0015 PMC3390115

[21] Greiner AN , Meltzer EO . Overview of the treatment of allergic rhinitis and nonallergic rhinopathy. Proc Am Thorac Soc 2011; 8:121–131. 21364230 10.1513/pats.201004-033RN

[22] Chen Y . Efficacy of sodium cromoglicate eye drops combined with yupingfeng granules in the treatment of allergic conjunctivitis. Eye Sci 2013; 28:201–203. 24961093

[23] Xing D , Zhang R , Li S *et al* Pivotal role of mast cell carboxypeptidase A in mediating protection against small intestinal ischemia‐reperfusion injury in rats after ischemic preconditioning. J Surg Res 2014; 192:177–186. 24953986 10.1016/j.jss.2014.05.050

[24] Stevens M , Edwards A . The effect of 4% sodium cromoglicate cutaneous emulsion compared to vehicle in atopic dermatitis in children ‐ a meta‐analysis of total SCORAD Scores. J Dermatolog Treat 2014; 18:1–7. 10.3109/09546634.2014.933766 24916212

[25] Han D , Hu Y , Li L *et al* Highly pathogenic porcine reproductive and respiratory syndrome virus infection results in acute lung injury of the infected pigs. Vet Microbiol 2014; 169:135–146. 24472226 10.1016/j.vetmic.2013.12.022 PMC7127595

[26] Hu Y , Jin H , Du X *et al* Effects of chronic heat stress on immune responses of the foot‐and‐mouth disease DNA vaccination. DNA Cell Biol 2007; 26:619–626. 17688414 10.1089/dna.2007.0581

[27] Hoffmann E , Stech J , Guan Y , Webster RG , Perez DR . Universal primer set for the full‐length amplification of all influenza A viruses. Arch Virol 2001; 146:2275–2289. 11811679 10.1007/s007050170002

[28] Monteerarat Y , Sakabe S , Ngamurulert S *et al* Induction of TNF‐alpha in human macrophages by avian and human influenza viruses. Arch Virol 2010; 155:1273–1279. 20532927 10.1007/s00705-010-0716-y

[29] Zhou J , Law HK , Cheung CY , Ng IH , Peiris JS , Lau YL . Differential expression of chemokines and their receptors in adult and neonatal macrophages infected with human or avian influenza viruses. J Infect Dis 2006; 194:61–70. 16741883 10.1086/504690 PMC7110244

[30] Nakajima N , Van Tin N , Sato Y *et al* Pathological study of archival lung tissues from five fatal cases of avian H5N1 influenza in Vietnam. Mod Pathol 2013; 26:357–369. 23174938 10.1038/modpathol.2012.193 PMC9813393

[31] Marjuki H , Mishin VP , Chesnokov AP *et al* Characterization of Drug‐Resistant Influenza A(H7N9) Variants Isolated From an Oseltamivir‐Treated Patient in Taiwan. J Infect Dis 2015; 211:249–257. 25124927 10.1093/infdis/jiu447 PMC6943751

[32] Samson M , Abed Y , Desrochers FM *et al* Characterization of Drug‐Resistant Influenza Virus A(H1N1) and A(H3N2) Variants Selected *In Vitro* with Laninamivir. Antimicrob Agents Chemother 2014; 58:5220–5228. 24957832 10.1128/AAC.03313-14 PMC4135884

[33] Grund S , Gkioule C , Termos T *et al* Primarily oseltamivir resistant influenza A (H1N1pdm09) virus evolving into a multi‐drug resistant virus carrying H275Y and I223R neuraminidase substitutions. Antivir Ther 2014; 20:90–100. 10.3851/IMP2811 24941247

[34] Yang YE , Wen J , Zhao S , Zhang K , Zhou Y . Prophylaxis and therapy of pandemic H1N1 virus infection using egg yolk antibody. J Virol Methods 2014; 206:19–26. 24880066 10.1016/j.jviromet.2014.05.016

[35] Hurt AC . The epidemiology and spread of drug resistant human influenza viruses. Curr Opin Virol 2014; 8C:22–29. 24866471 10.1016/j.coviro.2014.04.009

[36] Canini L , Conway JM , Perelson AS , Carrat F . Impact of different oseltamivir regimens on treating influenza A virus infection and resistance emergence: insights from a modelling study. PLoS Comput Biol 2014; 10:e1003568. 24743564 10.1371/journal.pcbi.1003568 PMC3990489

[37] Kwon D , Shin K , Kim SJ , Lee JY , Kang C . Mammalian pathogenesis of oseltamivir‐resistant pandemic (H1N1) 2009 influenza virus isolated in South Korea. Virus Res 2014; 185:41–46. 24657788 10.1016/j.virusres.2014.03.014

[38] Zhang A , Chi X , Luo G *et al* Mast cell stabilization alleviates acute lung injury after orthotopic autologous liver transplantation in rats by downregulating inflammation. PLoS ONE 2013; 8:e75262. 24116032 10.1371/journal.pone.0075262 PMC3792971

[39] Hayashi T , Hiromoto Y , Chaichoune K *et al* Host cytokine responses of pigeons infected with highly pathogenic Thai avian influenza viruses of subtype H5N1 isolated from wild birds. PLoS ONE 2011; 6:e23103. 21826229 10.1371/journal.pone.0023103 PMC3149639

[40] Cilloniz C , Pantin‐Jackwood MJ , Ni C *et al* Lethal dissemination of H5N1 influenza virus is associated with dysregulation of inflammation and lipoxin signaling in a mouse model of infection. J Virol 2010; 84:7613–7624. 20504916 10.1128/JVI.00553-10 PMC2897611

[41] Aldridge JR , Moseley CE , Boltz DA *et al* TNF/iNOS‐producing dendritic cells are the necessary evil of lethal influenza virus infection. Proc Natl Acad Sci U S A 2009; 106:5306–5311. 19279209 10.1073/pnas.0900655106 PMC2664048

[42] Nelli RK , Dunham SP , Kuchipudi SV *et al* Mammalian innate resistance to highly pathogenic avian influenza H5N1 virus infection is mediated through reduced proinflammation and infectious virus release. J Virol 2012; 86:9201–9210. 22718824 10.1128/JVI.00244-12 PMC3416141

[43] Brown MG , McAlpine SM , Huang YY *et al* RNA sensors enable human mast cell anti‐viral chemokine production and IFN‐mediated protection in response to antibody‐enhanced dengue virus infection. PLoS ONE 2012; 7:e34055. 22479521 10.1371/journal.pone.0034055 PMC3316603

[44] Fukuda M , Ushio H , Kawasaki J *et al* Expression and functional characterization of retinoic acid‐inducible gene‐I‐like receptors of mast cells in response to viral infection. J Innate Immun 2013; 5:163–173. 23171655 10.1159/000343895 PMC6784038

[45] Orinska Z , Bulanova E , Budagian V , Metz M , Maurer M , Bulfone‐Paus S . TLR3‐induced activation of mast cells modulates CD8 + T‐cell recruitment. Blood 2005; 106:978–987. 15840693 10.1182/blood-2004-07-2656

